# Retrospective analysis of apical prolapse correction by unilateral pectineal suspension: perioperative and short-term results

**DOI:** 10.1007/s00192-023-05479-4

**Published:** 2023-02-14

**Authors:** Dimitrios Ilias Bolovis, Michael Schreibmayer, Wolfgang Hitzl, Cosima Veronika Maria Brucker

**Affiliations:** 1 University Women’s Hospital, Paracelsus Medical University, Nuremberg, Germany; 2 Georg Simon Ohm Technical University, Nuremberg, Germany; 3 Research Program Experimental Ophthalmology and Glaucoma Research, Paracelsus Medical University, Salzburg, Austria; 4 Department of Research and Innovation, Paracelsus Medical University, Salzburg, Austria; 5 Department of Obstetrics and Gynecology, Klinikum Nuremberg, Prof.-Ernst-Nathan-Str. 1, 90419 Nuremberg, Germany; 6 Barmherzige Brüder Krankenhaus, St. Veit/Glan, Austria; 7 Department of Ophthalmology and Optometry, Paracelsus Medical University, Salzburg, Austria

**Keywords:** Pelvic organ prolapse, Unilateral pectineal suspension, Robotic surgery

## Abstract

**Introduction and hypothesis:**

We have previously published the novel method of unilateral pectineal suspension (UPS) for apical prolapse correction. UPS provides mesh-free midline uterus suspension using a single non-absorbable suture to attach the anterior cervix to the lateral part of the iliopectineal ligament. The purpose of this retrospective cohort study was to analyze the short-term efficacy, perioperative complication rate, and overall patient acceptance of the new UPS surgical concept.

**Methods:**

Forty-seven patients with POP-Q stage 2–4 who underwent robotic UPS between January 1, 2020 and December 31, 2021 were included in the study. Patient data were taken retrospectively from the patient files. Treatment success was the primary endpoint, measured both objectively using a defined composite endpoint and subjectively according to patients’ acceptance 3–6 months after surgery during a follow-up examination. Secondary outcome measures included complications and conversions, and effect of additional procedures on operative time.

**Results:**

Treatment success as measured by the defined composite endpoint was 93.6% for the entire cohort. No complications or conversions occurred. Mean operation time for isolated UPS was 46.5 min (*n* = 33 patients). UPS can be easily combined with additional surgical procedures for repair of remaining pelvic floor defects, incontinence surgery or other indications. Additional procedures performed had a significant influence on operation time (*p *< 0.0005, *n* = 14).

**Conclusions:**

UPS shows highly favorable results when looking at an unselected cohort of patients in need of primary POP surgery with respect to established quality parameters of POP repair.

## Introduction

Pelvic organ prolapse (POP) has a prevalence of 3–6% when defined by symptoms and up to 50% when based upon vaginal examination in parous women [1]. Typical symptoms of pelvic floor dysfunction are intravaginal bulging, pelvic discomfort, urinary incontinence, and bowel disorders [2, 3]. Moreover, POP leads to a reduction in quality of life and psychosocial well-being [4].

The initial treatment of POP is frequently nonsurgical; however, most women will eventually ask for a surgical solution. The aim of surgical correction is the restoration of pelvic floor functional anatomy. The lifetime-risk of women undergoing surgery for POP repair has been estimated to be 11%, with a reoperation rate of 29% by the age of 79 [5, 6].

Sacrocolpopexy (SCP) has been the gold standard for treatment of apical and combined POP since the 1990s. SCP is normally combined with supracervical hysterectomy or else performed exclusively on the vaginal wall. The procedure morbidity in terms of chronic pelvic pain, constipation, defecation disorders, and urinary incontinence has been thoroughly evaluated [7–9]. Even in expert hands, SCP can be a challenging procedure associated with long-term pelvic floor dysfunctional results.

The German POP guideline working group has suggested that in the absence of uterine pathology, patient preference could present as an indication for uterine preservation. Established options are vaginal sacrospinous hysteropexy, abdominal sacrohysteropexy with mesh interposition, and fixation of the uterus to the sacrouterine ligaments [10]. As an alternative, Dubuisson lateral mesh suspension can offer simultaneous cystocele correction with uterus preservation [11].

In 2011, Bannerjee and Noe described laparoscopic pectopexy as a minimal invasive technique of prolapse surgery, especially for obese women [12]. Pectopexy uses the lateral parts of the pectineal ligament (Cooper ligament) for fixation at the S2 level. As opposed to SCP and sacrospinous colpopexy, the vaginal axis was found to be near-normal in patients who underwent lateral mesh suspension [13]. The results after pectopexy show a significant improvement in sexual function and quality of life [14]. The robotic version of mesh-based pectopexy was first introduced by Bolovis et al. using the da Vinci Xi surgical system [15].

The broad use of mesh for POP surgery has been increasingly questioned, and has been restricted in some countries. Therefore, it seems important to implement reliable mesh-free alternatives. On the basis of the principle to use a lateral fixation point within the pelvis, we have established unilateral pectineal suspension (UPS) as a novel technique for apical and combined prolapse repair fulfilling a broad range of quality criteria [16]. UPS provides mesh-free midline uterus suspension using a single non-absorbable suture to attach the anterior cervix to the lateral part of the iliopectineal ligament. The purpose of this retrospective study was to analyze the short-term efficacy, perioperative complication rate, and overall patient acceptance of the new UPS surgical concept.

## Materials and methods

The present cohort study was conducted at the maximum care University Women’s Hospital of Klinikum Nuremberg, Paracelsus Medical University in accordance with the ethical standards of the Declaration of Helsinki. The study was approved by the internal review board of the Paracelsus Medical University (IRB-2022–009).

The Pelvic Floor Center at University Women’s Hospital of Klinikum Nuremberg offers state-of-the-art POP surgery with a broad repertoire of abdominal and vaginal approaches, including robotic surgery. Between January 1, 2020 and December 31, 2021, 47 patients presenting to our pelvic floor center with isolated or combined apical prolapse were scheduled for robotic UPS. All patients had failed standard options of conservative prolapse therapy and asked for surgical correction. Patients were informed on the surgical treatment options including the novel method of UPS. Exclusion criteria for robotic UPS were previous operations for prolapse correction and previously identified or strongly suspected massive adhesions in the abdominal cavity. Informed consent was obtained for robotic UPS, including the option of additional vaginal repair for residual defects during the same session. In cases of benign uterine pathology, patients were scheduled for a simultaneous supracervical hysterectomy.

All patients were preoperatively evaluated by a specialized team of pelvic surgeons. This included a problem-oriented history and a specialized vaginal examination to evaluate the status of the pelvic floor according to IUGA/ICS [17]. All patients presented with a minimum apical prolapse of POP-Q stage 2. Patients with symptoms of incontinence received an urodynamic evaluation.

**Fig. 1:**
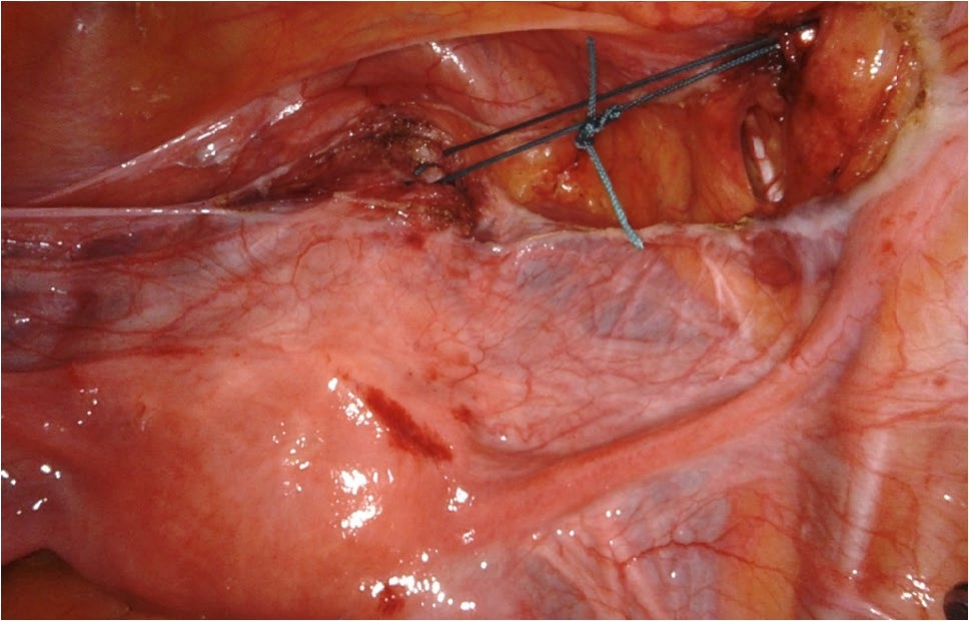
Intraoperative demonstration of UPS, showing the relationship of the pectineal ligament, non-absorbable stitch and anterior cervix

Surgery was carried out under standard general anesthesia. All cases were performed by the same team of two robotic and pelvic floor surgeons with ample experience in both disciplines, using the da Vinci Xi® surgical system. Determination of pre-operative POP-Q stage was carried out during vaginal exam at the beginning of the procedure. Simulation of the optimal position of the uterus was used to determine the desired final result. UPS was conducted as previously described in five defined steps [16]. The main anatomical landmarks for UPS are the lateral part of the pectineal (Cooper) ligament at the S2 level and the anterior cervix at the isthmo-cervical transition. An ethibond #2 suture is placed through the pectineal ligament, and subsequently attached to the anterior isthmo-cervical transition with three to four deep running parenchymal stitches. The uterus is guided to its desired position by two rotunda vulsellum forceps. The suture is adjusted to keep the uterus suspended at the ideal final position, and tied (Fig. 1). The peritoneum is closed with a vicryl suture. As a result of UPS, the uterus is positioned in midline anatomical position, thereby restoring the natural vaginal axis and preserving physiologic pelvic floor mobility.

After completion of robotic UPS, patients were re-examined vaginally to determine UPS result and to determine any remaining pelvic floor defects. If clinically indicated, remaining defects including cystocele, rectocele, or perineal insufficiency were corrected with anterior repair, posterior repair, and perineoplasty during the same session.

### Main outcome measures

Treatment success was defined as the primary endpoint, measured both subjectively (patient’s view) by a simple yes or no answer and objectively using a composite endpoint. The composite endpoint was determined as previously published, including anatomical and subjective components as well as the necessity for repeated surgery due to POP recurrence [18], as follows:

- (1) A POP-Q stage of ≤ 1 for the leading edge of the anterior or apical vaginal wall,
- (2) Absence of a vaginal bulge symptom and
- (3) No need for repeated surgery for anterior or apical POP.

For assessment, patients were scheduled for a follow-up visit 3 to 6 months after surgery.

### Secondary outcome measures

For secondary outcome measures, all patients were analyzed in terms of estimated blood loss in g/dl (EBL, calculated as the difference between hemoglobin level at admission versus hemoglobin level on the first postoperative day), operation time in minutes (i.e., the time when surgical measures were carried out starting with vaginal examination and ending with the last suture applied), necessity of conversion, and postoperative complications as detected perioperatively during hospital stay or indicated by readmission within the following 2 weeks. Docking and undocking of the da Vinci Xi® robotic system was included in the specified operation time, since these processes were carried out after vaginal examination and abdominal skin incision and before final skin closure. Setting up the robot including arm draping was performed before vaginal examination took place, and thus was not included in the specified operation time. BMI in kg/m^2^ was calculated by standard formula for all patients, and the influence of BMI on operation time was analyzed.

Patient data were collected retrospectively from the patient files. Postoperative ultrasound of bladder and kidneys was routinely performed on the first post-operative day to rule out urinary obstruction or retention.

A subjective overall satisfaction assessment was taken with a simple yes or no question on the day of the routine follow-up visit 3 to 6 months after surgery. Furthermore, patients were routinely asked for symptoms of stress or urge incontinence or any obstructive bowel symptoms.

### Statistical methods

Data were checked for consistency and normality. Fisher’s exact test or Pearson’s test were used to analyze cross tabulations. Repeated measures ANOVA, Student *t*-test with and without the assumption of variance homogeneity were used to test normally distributed variables. Levene’s test was used to test variance homogeneity. Fisher’s least significant difference tests were used for pairwise comparisons. Generalized linear models with log Gamma distribution were used for time of surgery; 95% CI were computed for means and illustrated using whisker plots. All reported tests were two-sided, and *p*-values < 0.05 were considered statistically significant. All statistical analyses in this report were performed by using STATISTICA 13 [19].

## Results

### Demographic patient data

Over a period of 24 months, a total of 47 consecutive patients met the inclusion criteria for participation in this retrospective study. Mean patient age was 64.6 years (range 35–84, SD 13). Mean patient weight was 64.4 kg with a mean BMI of 24.5 kg/m^2^ (range 17.8–43.2, SD 4.5). The majority of women were parous with at least one vaginal delivery (range 1–6 vaginal deliveries, mean 2.1, SD 1.2); only four women were non-parous (8.5%). Within the subgroup of women who had had vaginal deliveries, there was only one assisted delivery, and this was in a woman who also had one spontaneous vaginal birth. All other deliveries were spontaneous vaginal births. There was one C-section performed in the entire cohort, and this patient had also had one vaginal delivery (Table 1). All patients in the cohort underwent isolated or combined UPS.

**Table 1:** Demographic data

Characteristic	Value
Age (years)	64.6 ± 13 (range 35–84)
Body mass index (kg/m^2^)	24.5 ± 4.5 (range 17.8–43.2)
Parity: *n* (%)	
0	4 (8.5%)
1	6 (12.7%)
2	23 (48.9%)
≥ 3	14 (29.8%)
POP-Q stage (leading edge): *n* (%)	
II	11 (23.4%)
III	26 (55.3%)
IV	10 (21.3%)

Demographic data are presented as follows: Patient age in years (range), BMI in kg/m^2^ (range), Parity in number of patients with the indicated parity (percent of entire cohort), POP-Q stage of the leading edge in number of patients with the indicated POP-Q stage (percent of entire cohort)

Patient characteristics (*n* = 47)

### Surgical procedures

Of the 47 patients in this cohort, 33 patients (70.2%) were treated by isolated UPS without any additional surgical measures, while in the remaining 14 cases 15 additional procedures were performed (Table 2). These additional procedures were either indicated and planned preoperatively due to specific pathology, or performed intraoperatively to correct remaining pelvic floor defects after completion of UPS.

**Table 2:** Additional surgical procedures

Robotic procedures	*N*	Vaginal procedures	*N*
Supracervical hysterectomy*	3 (6.4%)	Perineoplasty*	7 (14.9%)
Burch colposuspension	2 (4.3%)	Mini-sling	2 (4.3%)
Robotic rectopexy	1 (2.1%)		

^*^One patient received both a supracervical hysterectomy and a perineoplasty

The cases with pre-planned procedures included three patients (6.4%) who underwent supracervical hysterectomy due to uterine fibroids, and four patients (8.5%) presenting with pronounced stress urinary incontinence as verified by urodynamic examination. These patients received either a vaginal mini-sling (*n* = 2) or Burch colposuspension (*n * = 2). In one patient, robotic mesh-free rectopexy was performed in addition to UPS.

Seven patients (14.9%) received perineoplasty due to a wide hiatus and introitus in addition to UPS and following intraoperative assessment.

### Treatment success

To examine treatment success as our primary endpoint, patients were re-evaluated during a follow-up visit 3 to 6 months after surgery (median 134 days, range 85 days). Subjective satisfaction and clinical findings were recorded. The participation rate upon invitation for follow-up re-examination was 100%. Using a simple yes or no format, 44 out of 47 patients (93.6%) stated that they were satisfied with the post-operative result.

The anatomical results were assessed using the POP-Q criteria as specified in the Materials and methods section. The examination showed stage ≤ 1 at the apex in 44 of the 47 patients, and treatment success according to the primary composite endpoint was thus achieved in 93.6% of the cases. Prolapse symptoms were reported by three of the 47 patients. These patients presented with leading cystocele stage 2 and complaints of intravaginal bulging. There was no relapse detected at the apex. Initial findings of those three patients had not been different from the other patients in the study, and no additional risk factors could be detected (not shown). Repeated surgery was performed in all three patients by vaginal repair.

### Complications and conversions

Within the 47 cases reported, no conversions were necessary. No intraoperative complications occurred, in particular no organ, vessel, or nerve injury or blood loss of > 200 ml. No drains were used. All patients received postoperative ultrasound of the kidneys and bladder to control for urinary obstruction and the ability to completely empty the bladder. All postoperative examinations were without finding. No complications directly related to the surgical procedure were observed in the perioperative phase. There were no postoperative procedure-related readmissions. Upon the follow-up visit, neither de-novo stress urinary incontinence or urgency nor obstructive bowel symptoms were noted in any of the patients treated with UPS. Furthermore, no vaginal or intra-abdominal discomfort was reported by any of the patients.

### Effects of additional procedures

**Fig. 2:**
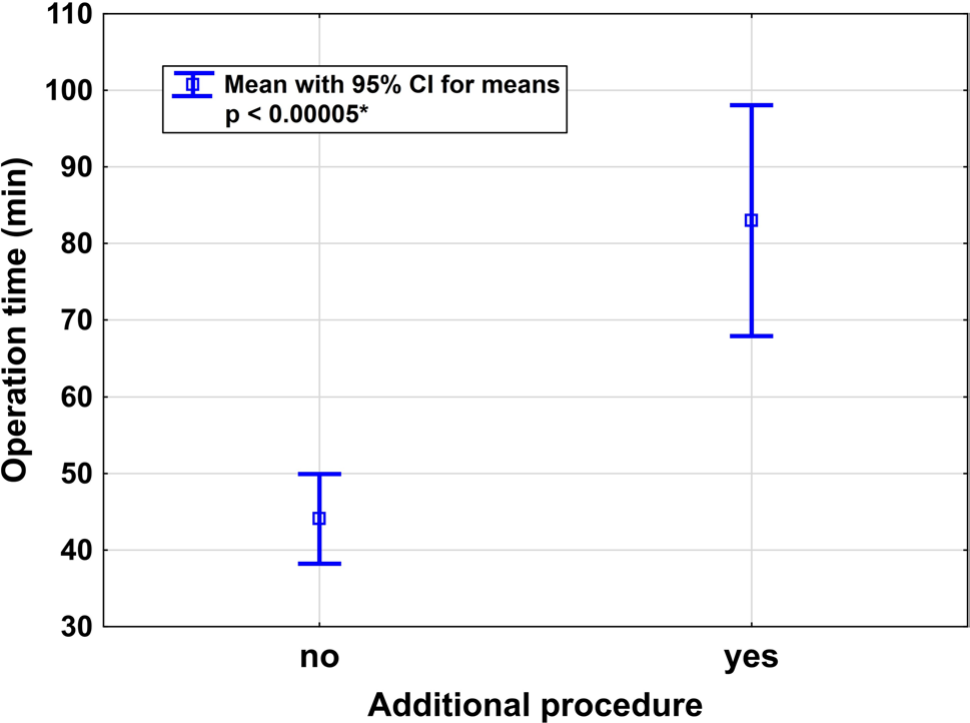
Operation time (min) with and without additional procedures

Additional procedures performed had a strong influence on operation time. Mean operation time for all cases was 61.7 min (SD 35.9, range 20–235, *n* = 47). Mean duration of surgery without additional procedures was 46.5 min (SD 13.5, range 20–74, *n* =  33), whereas mean duration of surgery with additional procedures was 97.6 min (SD 46.2, range 53–235, *n* =  4), and thus significantly longer (*p* < 0.00005, Fig. 2). The longest documented procedure was UPS with extensive adhesiolysis and Burch colposuspension (235 min). Within the group of patients who had additional procedures, there was no significant difference in total operation time whether the additional procedures were performed robotically versus vaginally (*p* = 0.17).

### Estimated blood loss

**Fig. 3:**
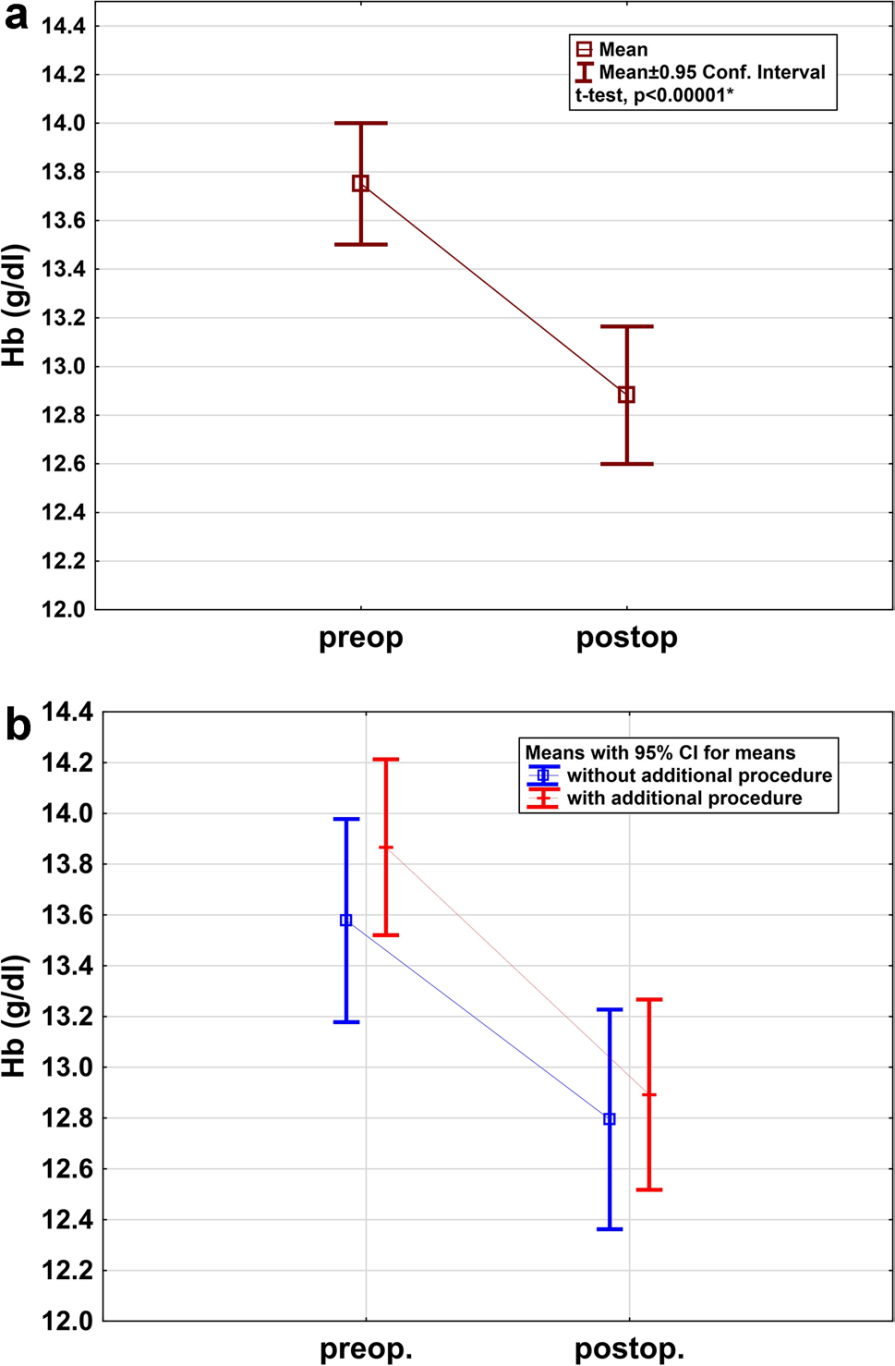
**a** Pre- and postoperative hemoglobin levels in the entire cohort. **b** Pre- and postoperative hemoglobin levels in patients with and in patients without additional procedures

Estimated blood loss was calculated from blood hemoglobin levels measured pre- and postoperatively. Mean hemoglobin levels were 13.71 g/dl (SD 0.84) before and 12.90 g/dl (SD 0.90) after surgery, resulting in a mean decrease of 0.81 g/dl (95% CI: 0.58–1.04) for the whole cohort (*p *< 0.00001, Fig. 3a). Mean hemoglobin levels in patients without additional procedures were 13.62 g/dl (SD 0.84) before and 12.86 g/dl (SD 0.97) after surgery, resulting in a mean decrease of 0.76 g/dl (95% CI: 0.48–1.04). Mean hemoglobin levels in patients with additional procedures were 13.92 g/dl (SD 0.83) before and 12.99 g/dl (SD 0.84) after surgery, resulting in a mean decrease of 0.93 g/dl (95% CI: 0.51–1.35). There was no significant difference in the degree of blood loss between the two groups (*p* = 0.50, Fig. 3b).

### Influence of BMI on operative time

**Fig. 4:**
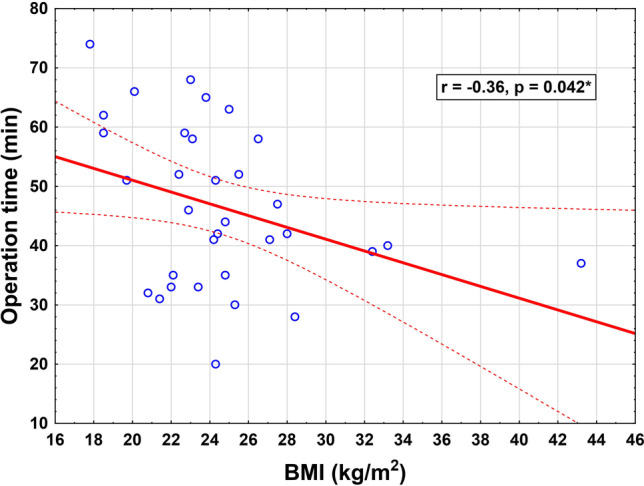
Correlation of BMI and operative time in patients with stand-alone UPS

BMI ranged from 17.8 to 43.2 kg/m^2^ and we found no correlation with operative time in patients with additional procedures (*r* = -−0.16, * p* = 0.59). Interestingly, a weak correlation was found towards a shorter operation time in patients with higher BMI in the group of patients without additional procedures (*r* = -−0.36, * p * = 0.04, Fig. 4), and this finding supports the advantages of the UPS concept in patients with higher BMI.

## Discussion

We have previously established UPS as a novel, minimally invasive, mesh-free suspension technique for isolated or combined apical POP correction in five defined steps [16]. The uterus can be preserved unless uterine pathology warrants hysterectomy. The clinical justification for this technique is based on the tension-free attachment of two broadly used and thoroughly analyzed anatomical landmarks of POP surgery: the pectineal ligament and the anterior cervix. As opposed to bilateral fixation we intentionally chose unilateral suspension since it avoids tension on the suspended structures and allows physiologic mobility. The procedure respects the physiological direction and angulation of the vaginal axis by repositioning the uterus to its original place within the lesser pelvis.

In order to further examine the efficiency and safety of UPS during routine clinical application, we have retrospectively analyzed perioperative parameters and short-term treatment success after 3–6 months in a cohort of 47 women presenting with apical prolapse POP-Q stage 2 or higher, including stage 4 total prolapse with vaginal inversion. We also included patients with additional pre-planned surgical procedures for associated urogynecological indications such as stress urinary incontinence and rectum prolapse, as well as necessary supracervical hysterectomy due to enlarged uterus. Furthermore, patients with an intraoperative indication for additional vaginal correction of remaining pelvic floor defects after completion of UPS were included. Thus, a real-world pattern of individual settings for POP repair is depicted by this study. Our data show that reliable apical fixation is effectively accomplished by UPS in all POP stages, including stage 4 total prolapse. The vast majority of patients was satisfied by the result of their POP correction. Furthermore, UPS is showing excellent perioperative characteristics. It can be combined conveniently with additional surgical procedures.

We had defined treatment success as our primary outcome measure. When examining the outcome after prolapse surgery, it is important to consider both the anatomical and the subjective results. We applied the POP-Q criteria in the setting of a composite endpoint of anatomical and subjective components including the necessity for repeated surgery due to POP recurrence as previously described [13]. In our case series, short-term anatomic outcome stayed within the definition of a successful repair in 93.6%. There were no apical recurrences, and thus apical correction was 100% successful. Forty-four of 47 patients reported their satisfaction with the operative result in a standardized follow-up interview. Three patients needed repeated surgery due to symptomatic grade 2 cystocele upon follow-up examination.

When comparing UPS with other approaches for hysteropexy, our data show an excellent outcome. For example, a 92% composite endpoint success was achieved following mesh-free anterior sacrospinous hysteropexy at 7.6 months follow-up [20]. In a prospective analysis of transvaginal mesh hysteropexy, an 84% composite endpoint success was reported at a median follow-up of 12 months [21]. Finally, short-term outcome after laparoscopic lateral suspension with mesh showed anatomic success rates of 88.2% for the anterior, 86.1% for the apical, and 80.8% for the posterior compartment after 12 months [22].

Extensive search of the international literature has shown that apical POP correction can be performed using various different approaches including SCP, vault suspensions, sacrospinous fixation, and vaginal obliterative procedures [23, 24]. In recent guidelines and reviews, the use of the pectineal ligament as a fixation structure for POP repair has not yet been adopted. Nevertheless, pectineal ligament suspension has been successfully conducted in incontinence surgery such as Burch colposuspension for over 50 years, and has been analyzed thoroughly [25, 26].

We have previously postulated intra-/perioperative and general quality criteria for surgical correction of pelvic organ prolapse (16, Table 3). Applying the proposed criteria to the different procedures for hysteropexy, we find that UPS fulfills every single criterion, while all other known options fail to do so in more than one category each. Furthermore, our retrospective data show that UPS was safely conducted without perioperative complications. Bladder and bowel function were not negatively affected by the procedure.

**Table 3:** Quality criteria for surgical correction of pelvic organ prolapse

Intra- /perioperative quality criteria	General quality criteria
Anatomical POP correction	No subsequent dyspareunia
Preservation of vaginal axis	Stable result
Uterine preservation	MIS approach
Preservation of natural pelvic floor mobility	Mesh-free
No vaginal tissue scarring	Fast procedure
Avoid bowel or ureter dissection	Day-case surgery

We used the da Vinci robotic system to perform UPS, since when using robotic technology, especially in pelvic floor reconstruction, operation times can be shortened as compared to conventional laparoscopy. This is due to a significant reduction of surgical compromise as well as the simplification of suture and knot application. Anatomical dissection and minimized blood-loss add to accuracy and surgical ergonomy. When performing UPS as a stand-alone procedure, mean operation time was 46.5 min using the da Vinci® robotic system. In contrast, even in experienced hands, classic techniques of POP repair such as SCP display operation times of approximately 180 min even when performed robotically [27, 28]. This difference underscores the quality aspects of UPS combining anatomical POP correction with a fast procedure. Certainly, the procedure is also suitable for laparoscopy or open abdominal surgery.

A weak correlation was found towards a shorter operative time in patients with higher BMI in the group of patients who had received UPS as a stand-alone procedure, while in the published literature robotic-assisted SCP showed no impact of BMI on operation time [29]. Thus, the UPS concept may be favorable also in obese patients. This aspect should be further examined in future studies.

Certainly, the study is limited by its retrospective design as well as the very short follow-up interval, making our data preliminary. We will re-analyze outcome data for our patient cohort at a later time-point to provide intermediate and long-term results. Prospective multicenter studies are under way to gain a broad data basis for the UPS concept.

In summary, short-term analysis of POP repair by UPS shows highly favorable results when looking at an unselected cohort of patients in need of primary POP surgery. UPS fulfills a large panel of quality criteria for POP surgery in a real-world setting, making it a strong candidate for a new standard of care.

## References

[1] Barber MD, Maher C (2013). Epidemiology and outcome assessment of pelvic organ prolapse. Int Urogynecol J.

[2] Bradley CS, Zimmerman MB, Wang Q, Nygaard IE (2008). Vaginal descent and pelvic floor symptoms in postmenopausal women. Obstet Gynecol.

[3] Slieker-ten Hove MCP, Pool-Goudzwaard AL, Eijkemans MJC, Steegers-Theunissen RPM, Burger CW, Vierhout ME (2009). Prediction model and prognostic index to estimate clinically relevant pelvic organ prolapse in a general female population. Int Urogynecol J.

[4] Ugurlucan FG, Evruke I, Yasa C, Dural O, Yalcin O (2020). Sexual functions and quality of life of women over 50 years with urinary incontinence, lower urinary tract symptoms and/or pelvic organ prolapse. Int J Impot Res.

[5] Olsen A, Smith V, Bergstrom J, Colling J, Clark A (1997). Epidemiology of surgically managed pelvic organ prolapse and urinary incontinence. Obstet Gynecol.

[6] Choi KH, Hong JY (2014). Management of pelvic organ prolapse. Korean J Urol.

[7] Baessler K, Schuessler B (2001). Abdominal sacrocolpopexy and anatomy and function of the posterior compartment. Obstet Gynecol.

[8] Culligan PJ, Murphy M, Blackwell L, Hammons G, Graham C, Heit MH (2002). Long-term success of abdominal sacral colpopexy using synthetic mesh. Am J Obstet Gynecol.

[9] Shiozawa T, Huebner M, Hirt B, Wallwiener D, Reisenauer C (2010). Nerve-preserving sacrocolpopexy: anatomical study and surgical approach. Eur J Obstet Gynecol Reprod Biol.

[10] Bässler K, Aigmüller T, Albrich, S et al. Diagnosis and treatment of the pelvic organ prolapse. Guideline of the German Society of Gynecology and Obstetrics (S2e-Level, AWMF Registry No. 015/006, April 2016). Geburtshilfe Frauenheilkd. 2016;76(12):1287–1301. 10.1055/s-0042-119648.

[11] Dubuisson J (2017). Patient satisfaction after laparoscopic lateral suspension with mesh for pelvic organ prolapse: outcome report of a continuous series of 417 patients. Int Urogynecol J.

[12] Banerjee C, Noé KG (2011). Laparoscopic pectopexy: a new technique of prolapse surgery for obese patients. Arch Gynecol Obstet.

[13] Pulatoglu C, Yassa M, Turan G, Türkyilmaz D, Dogan O. Vaginal axis on MRI after laparoscopic lateral mesh suspension surgery: a controlled study. Int Urogynecol J. 2020. 10.1007/s00192-020-04596-8. Online ahead of print. 10.1007/s00192-020-04596-8 33175232

[14] Tahaoglu AE, Bakir MS, Peker N, Bagli İ, Tayyar AT (2018). Modified laparoscopic pectopexy: short-term follow-up and its effects on sexual function and quality of life. Int Urogynecol J.

[15] Bolovis D, Hitzl W, Brucker C (2021). Robotic mesh-supported pectopexy for pelvic organ prolapse: expanding the options of pelvic floor repair. J Robot Surg.

[16] Bolovis DI, Brucker CVM (2022). Unilateral pectineal suspension A new surgical approach for apical correction of pelvic organ prolapse. Facts Views Vis Obgyn.

[17] Haylen BT, Maher CF, Barber MD (2016). An International Urogynecological Association (IUGA) / International Continence Society (ICS) joint report on the terminology for female pelvic organ prolapse (POP). Int Urogynecol J.

[18] Barber MD, Maher C (2013). Epidemiology and outcome assessment of pelvic organ prolapse. Int Urogynecol J.

[19] Hill T, Lewicki P (2006). Statistics: methods and applications.

[20] Plair A, Dutta R, Overholt TL, Matthews C (2021). Short-term outcomes of sacrospinous hysteropexy through an anterior approach. Int Urogynecol J.

[21] Khandwala S, Cruff J (2021). Prospective analysis of transvaginal mesh hysteropexy in the treatment of uterine prolapse. Int Urogynecol J.

[22] Veit-Rubin N, Dubuisson JB, Lange S, Eperon I, Dubuisson J (2016). Uterus-preserving laparoscopic lateral suspension with mesh for pelvic organ prolapse: a patient-centred outcome report and video of a continuous series of 245 patients. Int Urogynecol J.

[23] NICE Guidance. Urinary incontinence and pelvic organ prolapse in women: management: © NICE. BJU Int 2019;123(5):777–803. 10.1111/bju.14763. 10.1111/bju.14763 31008559

[24] No authors listed. Pelvic Organ Prolapse: ACOG Practice Bulletin, Number 214. Obstet Gynecol. 2019;34(5):e126-e142. 10.1097/AOG.0000000000003519. 10.1097/AOG.0000000000003519 31651832

[25] Veit-Rubin N, Dubuisson J, Ford A (2019). Burch colposuspension. Neurourol Urodyn.

[26] Sohlberg EM, Elliott CS (2019). Burch Colposuspension. Urol Clin North Am.

[27] Glass Clark S, Melnyk AI, Bonidie M, Giugale L, Bradley MS (2022). Operative time for minimally invasive sacrocolpopexy: comparison of conventional laparoscopy versus robotic platform. J Minim Invasive Gynecol.

[28] Callewaert G, Bosteels J, Housmans S, Verguts J, Van Cleynenbreugel B, Van der Aa F, De Ridder D, Vergote I, Deprest J (2016). Laparoscopic versus robotic-assisted sacrocolpopexy for pelvic organ prolapse: a systematic review. Gynecol Surg.

[29] Menzella D, Thubert T, Joubert M, Lauratet B, Kouchner P, Lefranc JP (2013). Influence of body mass index on the outcomes of robotic-assisted laparoscopic sacrocolpopexy: a comparative retrospective study. Progress en Urologie.

